# STAT3 and STAT5 Targeting for Simultaneous Management of Melanoma and Autoimmune Diseases

**DOI:** 10.3390/cancers11101448

**Published:** 2019-09-27

**Authors:** Stella Logotheti, Brigitte M. Pützer

**Affiliations:** 1 Institute of Experimental Gene Therapy and Cancer Research, Rostock University Medical Center, 18057 Rostock, Germany; Styliani.Logotheti@med.uni-rostock.de; 2 Department Life, Light & Matter, University of Rostock, 18059 Rostock, Germany

**Keywords:** melanoma, autoimmune disease, inflammation, STAT3, STAT5, immunotherapy, tumor–immune cell interactions

## Abstract

Melanoma is a skin cancer which can become metastatic, drug-refractory, and lethal if managed late or inappropriately. An increasing number of melanoma patients exhibits autoimmune diseases, either as pre-existing conditions or as sequelae of immune-based anti-melanoma therapies, which complicate patient management and raise the need for more personalized treatments. STAT3 and/or STAT5 cascades are commonly activated during melanoma progression and mediate the metastatic effects of key oncogenic factors. Deactivation of these cascades enhances antitumor-immune responses, is efficient against metastatic melanoma in the preclinical setting and emerges as a promising targeting strategy, especially for patients resistant to immunotherapies. In the light of the recent realization that cancer and autoimmune diseases share common mechanisms of immune dysregulation, we suggest that the systemic delivery of STAT3 or STAT5 inhibitors could simultaneously target both, melanoma and associated autoimmune diseases, thereby decreasing the overall disease burden and improving quality of life of this patient subpopulation. Herein, we review the recent advances of STAT3 and STAT5 targeting in melanoma, explore which autoimmune diseases are causatively linked to STAT3 and/or STAT5 signaling, and propose that these patients may particularly benefit from treatment with STAT3/STAT5 inhibitors.

## 1. Introduction

Within the last decade, the cancer field has witnessed a rapid paradigm shift from traditional chemotherapy to immunotherapy. A major advantage of immunotherapeutics is that, instead of directly targeting and killing the tumor as cytotoxic drugs do, they stimulate a person’s own immune system to recognize and destroy cancer cells. In this respect, they ally with the immune cells not only to shrink the primary tumor but also to establish durable responses against circulating cancer cells that might lurk beyond the primary site. Cancer immunotherapy approaches include (a) checkpoint inhibitors, which act by releasing the brakes that prevent T-cells from killing cancer cells, (b) monoclonal antibodies, that are designed to attach to cancer cell-specific antigens, (c) cancer vaccines, boosting the immune system’s response to tumors, or (d) cell-based therapies, such as chimeric antigen receptor (CAR) T-cell therapy, where T cells taken from a patient’s tumor are expanded and/or genetically engineered ex vivo and then administered back to the patient, a process termed adoptive transfer [1]. The field of successful application of immunotherapy includes, but is not limited to, metastatic melanoma [2], a type of cancer that arises from melanocytes and represents the deadliest form of skin cancer, with increasing prevalence. Once it becomes metastatic, the prognosis is very unfavorable and, thus, early diagnosis is crucial for effective management [3].

Since 2011, several next-generation immune-based formulations, such as the checkpoint inhibitors ipilimumab, pembrolizumab, and nivolumab, received approval by the Food and Drug Administration (FDA) for the indication of metastatic melanoma [2] and led to significant clinical improvements, either as monotherapies or in combination regimens [4]. However, despite their remarkable success, only up to 20% to 30% of patients have benefited from these treatments, while the rest are either non-responders (primary resistance) or partial responders (acquired resistance). Unresponsiveness is attributed to factors such as CD8+ T cell density in the tumor microenvironment, monocyte frequency, tumor heterogeneity, and neoantigen load, as well as the composition of patient’s gut microbiota [4].

Other challenges regarding immunotherapy include immune-related toxicities. In particular, 85% of melanoma patients under ipilimumab treatment have experienced immune-related adverse events of any grade, with over one-third discontinuing therapy or requiring additional systemic treatment to manage side effects [5]. Immunotherapy faces limitations in patients with both an overactive (autoimmune disease patients) or a suppressed (organ transplant recipients) immune system. On the one hand, melanoma patients with pre-existing autoimmune diseases who receive ipilimumab treatment present frequent disease flares and exacerbations, requiring additional immunosuppression or therapy discontinuation [6,7]. On the other hand, solid organ transplant recipients are at increased risk of developing metastatic melanoma, and when they do, they exhibit a higher probability for graft rejection upon immune checkpoint inhibitor treatment [8,9]. In general, a dysregulated immune system poses as the Sword of Damocles in the decision of clinicians to prescribe immunotherapy. In this regard, there is an increasing need to develop drugs for the treatment of melanoma that are not only safer for such patients but are also able to manage this cancer along with a co-existing immune-related disease.

Herein, we hypothesize that therapeutic management of various clinical disorders simultaneously can be achieved by targeting their major common pathways. As a representative case, we consider the Janus kinase/signal transducers and activators of transcription (JAK/STAT) pathway, which is activated in a wide range of autoimmune diseases (AD), as well as in many different cancer types. It mediates immune responses to several insults from resisting infection to maintaining immune tolerance, enforcing barrier functions, and guarding against cancer [10]. In this review, we summarize ADs that are associated with malignant melanoma, we explore how STAT3 and STAT5 signaling contributes to their pathogenesis, and we evaluate STAT3/STAT5 inhibition as a feasible strategy to target these diseases in order to achieve their simultaneous management with a single drug.

## 2. Associations among Melanoma, Inflammation and Autoimmune Diseases

**Figure 1:**
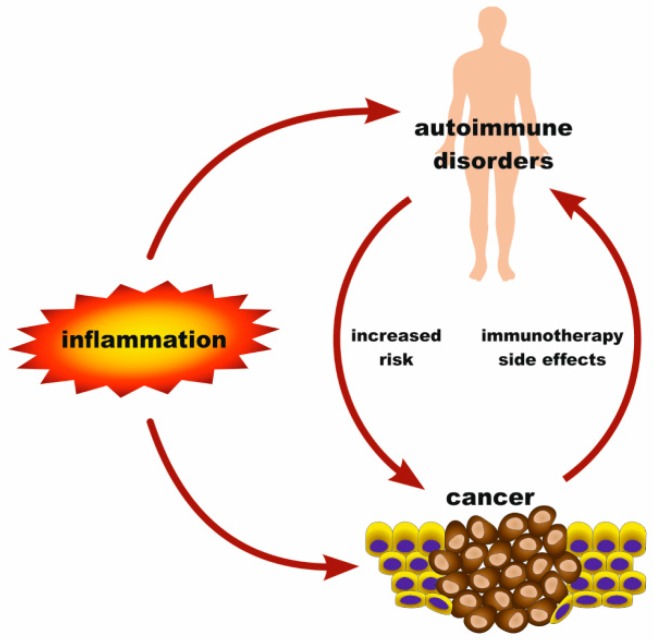
Associations between inflammation, cancer, and autoimmune disease. An inflammation can eventually lead to cancer or autoimmune disease. Some autoimmune disorders may predispose to malignancies. Cancer immunotherapy is associated to autoimmunity.

A cancer patient can frequently experience disorders, such as inflammation and autoimmune diseases, that occur as frequent pre-existing, predisposing, or intercurrent conditions [11,12,13]. Intriguingly, these diseases seem to be etiologically interrelated with one another. On one side, cancer has been linked with chronic inflammation. Moreover, there are interconnections between autoimmune diseases and cancer, since certain autoimmune disorders predispose to neoplasias [14]. Patients suffering from dermatomyositis, inflammatory bowel disease, systemic lupus erythematosus, rheumatoid arthritis, psoriasis or Sjögren syndrome may have increased risks for malignancies, whereas the type(s) of cancer they tend to develop depend on the type of the autoimmune disease [15]. For example, rheumatoid arthritis patients are at a greater risk of developing clonal expansions of large granular lymphocytes [16]. Although an autoimmune disorder does not necessarily lead to cancer, it is, however, a phenotypic manifestation of a deregulated immune system, which is generally a favorable background for cancer development [17]. Vice versa, cancer immunotherapy triggers autoimmunity towards several anatomical sites and can lead to conditions that range from relatively minor, such as skin depigmentation, to severe colitis, pancreatitis, lung or liver toxicity [18]. Furthermore, a persistent inflammation can be a fertile ground for the development of an AD [19]. Overall, an inflammation can potentially progress to a neoplasia or an autoimmune disorder, and this process can be facilitated by deregulation of the innate immune system [11,14] (Figure 1).

This interplay among ADs and cancer is evident in the case of melanoma patients. First of all, there is a long-standing inverse association between melanoma and vitiligo, a condition where the immune system produces autoantibodies against immunogenic, melanocytic-specific molecules (melanocytic-differentiation antigens, MDAs) that propel melanin production. These autoantibodies attack melanocytes, ultimately producing white skin patches. Thus, vitiligo patients show a lower melanoma risk because the antibodies against melanocytes confer natural cancer protection [20]. Vice versa, some melanoma patients develop a similar antibody-based condition, called melanoma-associated hypopigmentation or vitiligo-like depigmentation, which is usually considered as a predictor of better outcomes [21], although there are also reports demonstrating that hypopigmentation can be associated with disease progression [22,23,24]. Given that malignant melanoma co-evolves with immune cell phenotypes [25], this dynamic interaction between autoimmunity-based hypopigmentation and skin cancer could, in the long run, become a double-edged sword. Specifically, these tumors exhibit a high intratumoral heterogeneity and plasticity [25]. Therefore, over the course of the disease, tight immunosurveillance against MDA-expressing cells could serve as a microenvironmental cue that adds evolutionary pressure for immunoselection of low-MDA expressing cell variants that are poorly recognized by the autoantibodies. Such MDA-negative cells possess enhanced invasive capabilities. In this way, anti-MDA responses provoked by the melanoma tumor, can, in turn, promote clonal expansion of low-MDA-expressing cell variants with activated prometastatic programs that can migrate to distant sites, giving rise to secondary tumors [24].

Other autoimmune diseases are positively correlated with melanoma progression [2,26]. A retrospective meta-analysis assessed that the prevalence of pre-existing AD in melanoma patients increased by 1.7-fold within a decade. Prevalence rates were higher in metastatic melanoma patients compared to primary, non-metastatic skin cancer patients or the general population, suggesting that a pre-existing AD could possibly favor metastatic progression. The most common ADs among metastatic melanoma patients were myositis, peripheral neuropathy, type 1 diabetes mellitus, rheumatoid arthritis, psoriasis, autoimmune pancreatitis, autoimmune aplastic anemia, relapsing polychondritis, Hashimoto’s encephalopathy, and inflammatory bowel disease. The authors suggested that disturbances in shared molecular or immune pathways could underlie increased susceptibility of AD patients to melanoma [2]. Another clinical study showed that only a small percentage of melanoma patients have a pre-existing AD, but in these patients, progression is faster and prognosis is worse. This subpopulation has a significantly shorter median overall survival and disease-free survival after first metastasis versus cases with a primary tumor, while poorer prognosis was independent of the side effects of AD treatment. Those patients with an antibody-mediated AD had a worse prognosis in relation to those with a T cell-related AD. [27]. Together, these data unveil a potential link between malignant melanoma and immune dysregulation, implying that a pre-existing autoimmune disease could be a predisposing factor for melanoma development and progression. If that is indeed the case, further experimental investigations are needed.

The relationship between inflammation and cancer is well established [28] and inflammation contributes to melanoma initiation and progression [25]. Upregulation of key cancer-related inflammation genes, such as the transcription factors nuclear factor ‘kappa-light-chain-enhancer’ of activated B-cells (NF-κB) and STAT3, as well as several inflammatory cytokines and angiogenic factors, facilitate melanoma development. Indeed, inoculation of B16-F10 melanoma cells in chronic skin inflammation mouse models led to the formation of larger volume tumors in comparison with the non-inflamed controls, followed by increased periostin expression and M2 macrophage recruitment in the tumor microenvironment (TME) [29]. Similarly, in a mouse model of melanoma, repetitive UV exposure promoted metastasis via neutrophilic skin inflammation. In particular, UV irradiation-induced neutrophil recruitment and activation, which was initiated by the release of high mobility group box 1 (HMGB1) from UV-damaged epidermal keratinocytes. The UV-induced neutrophilic inflammatory response stimulated angiogenesis and increased the number of lung metastases through modulation of melanoma–endothelial cell interactions [30]. The association between inflammation and melanoma is also supported by the fact that anti-inflammatory drugs contribute to melanoma prevention or treatment. Population-based case-control studies showed that continuous use of low-dose non-steroid anti-inflammatory drugs (NSAIDs), such as diclofenac, ibuprofen, and naproxen, reduced the incidence of skin cancer, even though in a sex-dependent manner [31]. Other studies showed that combining celecoxib, a cyclooxygenase COX-2 selective NSAID, with chemotherapeutics can be an effective strategy for melanoma treatment [32].

## 3. A Basis for the Development of Multipotent Drugs against Melanoma, Inflammation, and Autoimmune Disorders

**Figure 2:**
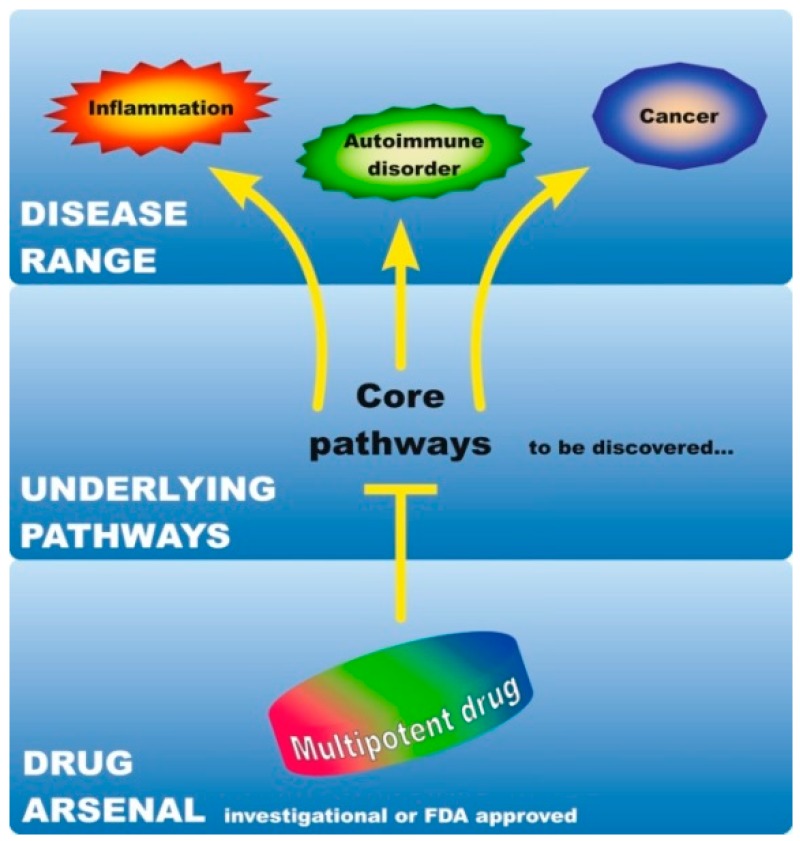
Proposed rationale for treating melanoma and associated inflammation or autoimmunity with multipotent drugs. Investigational or Food and Drug Administration (FDA)-approved drugs could exert anti-inflammatory, immunomodulatory, and anticancer effects. This, to an extent, might be due to their interference with signaling pathways commonly activated in inflammation, autoimmune disease, or cancer. These core pathways underlying all-three pathological conditions can be further characterized to facilitate simultaneous management of associated pathologies due to distinct diseases.

It is becoming increasingly evident that common molecular mechanisms underlie inflammatory-driven [12], as well as inflammatory/autoimmune-driven neoplasias [17]. The emerging similarities and interconnections among these diseases imply that despite their diverse clinical manifestations, outcome, and response to therapy, a limited number of mutual molecular events might drive their pathogenesis. In this regard, the mechanistic commonalities between cancer, inflammation, and ADs have created a basis for drug repurposing. For instance, anti-inflammatory drugs are used in combination with anticancer regimens [12,33]. Additionally, immunomodulatory drugs used for the treatment of ADs show efficacy against neoplastic diseases [34,35] and vice-versa, anticancer therapeutics can be applied to treat non-cancer immune-mediated diseases [36]. Recent network-based studies demonstrate that disorders with distinct clinical manifestations share a common genetic background [37]. Therefore, identifying shared signaling networks and cellular pathophenotypes at the core of these diseases is anticipated to further shape drug development in the future. We use the term ‘multipotent drug’ for any targeted inhibitor with the potential to act against two or more pathologies, even if these are different diseases, such as autoimmunity and cancer as an example. Moreover, given that they are mechanistically interrelated [11,12], we further postulate that the effectiveness of such a multipotent drug may depend on its interference with ‘root’ pathways that pose as common denominators in the pathology of these diseases. Hence, identification of these common traits is a prerequisite for the development of multipotent drugs, which could simultaneously manage cancer in conjunction with coexisting, pre-existing, or predisposing disorders (Figure 2).

To this end, considering that deregulation of immunity is the main culprit in inflammation, autoimmune disease, and melanoma, we hypothesized that pathways mediating immune responses might be suitable therapeutic targets. Given that the JAK/STAT pathway is commonly activated in these diseases, we postulate that it is an emerging candidate ‘core’ pathway. Herein, we review the current knowledge of the effects of STAT3 and STAT5 signaling in melanoma, inflammation, and autoimmune disorders and estimate their value for concurrent management of these interconnected conditions.

## 4. STAT3 and STAT5 Signaling in Melanoma Initiation and Progression

STAT proteins, particularly STAT3 and, to a lesser extent, STAT5 govern fundamental oncogenic processes. STAT family members respond to cytokines, growth factors, and hormones, and transduce signals from the cell membrane to the nucleus to transactivate genes involved in cellular immunity, proliferation, apoptosis, and differentiation. The STAT family encompasses seven members, STAT1, STAT2, STAT3, STAT4, STAT5A, STAT5B, and STAT6, which show high homology in their functional domains. Their main difference lies in the C-terminal transactivation domain that varies among family members and modulates transcriptional activation of target genes. Under physiologic conditions, STAT signaling is stimulus-dependent and tightly regulated. The regulation of STAT activity via phosphorylation of a conserved C-terminal tyrosine residue has been previously described in detail [38,39]. Constitutive activation of STAT3 and STAT5 is involved in tumor formation and progression. However, not all STAT members are promoting cancer progression. STAT1, for instance, exerts proapoptotic and antiproliferative effects, and it seems that both, STAT3 and STAT5 can antagonize its function [40].

STAT3 is constitutively activated across many types of human cancers, including melanoma, as a consequence of aberrant autocrine or paracrine stimulation by cytokines and growth factors, such as interleukins (IL-6, IL-10, IL-12), interferons (IFNs), granulocyte-colony stimulating factor (G-CSF or CSF3), leptin, prolactin (PRL), growth hormone (HGH), epidermal growth factor (EGF), hepatocyte growth factor (HGF), basic fibroblast growth factor (FGF2), and virus proteins (e.g., v-Src, v-Fps, v-Sis). Permanent STAT3 activation can also result from receptors with intrinsic tyrosine kinase activities (such as erb-b2 receptor tyrosine kinase 2 (ERBB2), epidermal growth factor receptor (EGFR), and hepatocyte growth factor receptor HGFR), non-receptor tyrosine kinases (such as c-Src and c-abl), or G-protein coupled receptors [38,41]. An overactive STAT3 is associated with poor prognosis in melanoma patients and drives tumor initiation and malignant progression via induction of several cancer hallmarks, such as apoptosis inhibition, tumor angiogenesis, epithelial–mesenchymal transition (EMT), and stemness. In particular, upon c-Src activation, STAT3 promotes tumor cell survival and proliferation by transactivating *BCL2L1* (that encodes the Bcl-XL protein) and *MCL1*, two crucial anti-apoptotic genes that override cell death regulatory pathways. Additionally, STAT3 regulates the *MYC* gene, which mediates escape of melanoma tumor cells from both terminal differentiation and G0/G1 arrest. Beyond its function as a transcriptional activator, STAT3 can also suppress transcription of the well-established tumor suppressor *TP53* [38]. This suppression can provide an explanation on why melanomas that lack mutations in either *TP53* or its main negative regulator, such as *MDM2,* exhibit aggressive characteristics. STAT3 upregulates nodal factors of angiogenesis, mainly vascular endothelial growth factor (VEGF), hypoxia-inducible factor 1a (HIF-1α) and matrix metalloproteinase-2 (MMP-2) [38] and fosters brain metastasis in melanoma [42]. In addition, drivers of melanoma invasion and metastasis, such as ∆Ex2/3p73 (named DNp73), a transactivation-deficient N-terminally truncated oncogenic isoform of the *TP73* gene, can trigger an IGF1R-AKT/STAT3 signaling cascade, which leads to the activation of EMT markers and acquisition of mesenchymal cell phenotypes [43,44]. Additionally, STAT3 is often mentioned in connection with ∆Np63 [45]. ∆Np63α indirectly drives STAT3 (Tyr705) phosphorylation in an autocrine loop by transactivating interleukin 6 (IL-6) and IL-8. Phosphorylated STAT3 stabilizes HIF-1α, resulting in an increased production of VEGF in ∆Np63α-overexpressing cells [44]. In melanoma, ∆Ex2/3p73, but not ∆Np63α, mediates STAT3 (Tyr705) phosphorylation in an EPLIN/IGF-1R-dependent manner [43]. With respect to its ability to promote cancer through regulating cancer stem cell (CSC) activities [46], STAT3 upregulation mediates reprogramming of melanoma cells to melanoma stem cells by inducing expression of the Yamanaka factors, providing hints of STAT3 implication in cancer stemness [47]. Moreover, STAT3 increases chemoresistance of melanomas to selective inhibitors of BRAF, which are exploited in the treatment of unresectable or metastatic melanoma with a *BRAF-V600* gain-of-function mutation [48].

Similar to STAT3, STAT5 also exerts oncogenic functions. There are two distinct genes, *STAT5A* and *STAT5B*, that have arisen by gene duplication and encode protein products which share more than 90% peptide sequence similarity and differ primarily in the C-terminus-encoding region [49]. STAT5A is predominantly expressed in the mammary gland, while STAT5B is prevalent in muscle and liver. They respond to a number of factors, such as prolactin, Growth Hormone, erythropoietin, thrombopoietin, EGF, IL-2, IL-3, IL-6, IL-7, IL-9, and IL-15 [40]. STAT5 promotes malignant transformation in hematological malignancies, breast and prostate cancer, non-small cell lung carcinoma, and melanoma [50]. The initial correlation between STAT5 activation and aggressive characteristics of melanoma was first described in a Xiphophorus fish melanoma model [51]. Subsequently, Hassel and colleagues showed that constitutive activation of STAT5 correlated with the upregulation of its antiapoptotic target gene *BCL2L1*, an effect which was conserved in both, human melanoma cells and murine melanocytes [52]. In melanoma patients, STAT5B transcripts were significantly upregulated, whereas STAT5 was found phosphorylated in 62% of the metastatic cases versus normal human melanocytes and benign nevi. STAT5 activation is induced by the EGF/JAK1 axis in melanoma cell lines. Knockdown of *STAT5B* in these cells causes downregulation of BCL2, which triggers cell death and G1 arrest, thus underscoring that STAT5B acts as a survival factor in melanoma [50]. In addition to its antiapoptotic role in melanoma, STAT5 influences the sensitivity to anti-melanoma treatment, including interferon alpha (IFN-α) immunotherapy and BRAF inhibitors. For instance, in skin cancer patients under adjuvant IFN-α therapy, STAT5 expression emerged as an independent predictor of progression-free survival. Recurrence in patients with STAT5-expressing tumors was observed either during or after IFN-α therapy [53]. In accordance herewith, STAT5 has been shown to be overexpressed in IFNα-resistant melanoma cells [54]. Moreover, this transcription factor regulates the nicotinamide phosphoribosyltransferase (NAMPT), a key enzyme in the maintenance of cellular nicotinamide adenine dinucleotide (NAD+) levels, in response to activated B-Raf/extracellular signal-regulated kinases (BRAF/ERK) signaling. NAMPT induces melanoma cell proliferation accompanied by a phenotypic switch towards an invasive phenotype and resistance to BRAF inhibitors [55]. Consistently, a combination of the neuroleptic drug pimozide with indoleamine 2,3-dioxygenase sensitizes melanoma cells to apoptosis and inhibits cell migration via STAT5 suppression [56]. However, pimozide is not a direct STAT5 inhibitor, but rather targets upstream pathway activation for degradation.

## 5. STAT3 and STAT5 in the Crosstalk between Melanoma and Immune Cells

Melanomas display qualitative and quantitative changes in the density, composition, functional state, and organization of immune infiltrates, the so-called immune contexture, which render immune cells tolerant to tumors or exhaust their ability to attack tumor cells [57]. STAT3 influences the interplay between melanoma cells and components of the immune system, thus contributing to immune evasion. Tumor and immune cells expressing STAT3 develop sophisticated interactions to overall support an immunosuppressive tumor environment that propels metastatic progression. In detail, melanoma cells which overexpress STAT3 protein inhibit the expression of proinflammatory cytokines and chemokines, such as VEGF, IL-10, and IL-6. These, in turn, induce STAT3 activity in hematopoietic progenitor cells (HPCs) to promote the production of immature myeloid cells (IMCs) and plasmacytoid dendritic cells (pDCs). Through IL-10, IMCs block the maturation of dendritic cells into antigen-presenting cells. Thus, these immature dendritic cells are unable to stimulate the antitumor effects of CD8+ T cells and natural killer (NK) cells. In parallel, pDCs promote the accumulation of regulatory T cells (Treg) in the tumor microenvironment. IL-10 and TGF-β secreted by Treg cells further enhance the immunosuppressive microenvironment by restraining both, CD8+ effector T-cell function and DC maturation [38,58,59,60]. Direct STAT3 targeting in melanoma cell lines sufficed to modulate these crosstalks and enhanced antitumor activity through increasing interferon gamma (IFNγ) levels, mature dendritic cells, and CD8+ T cells. This resulted in reduced tumor growth and prolonged survival of tumor-bearing mice [61]. There is the first evidence that STAT3 activation in NK cells that trigger innate immune responses by the destruction of the tumor cells, can suppress their cytotoxicity against melanoma cells in vivo. The dual effect of STAT3 in melanoma, as well as tumor-attacking immune cells, is of great therapeutic interest since STAT3 inhibition not only blocks survival of cancer cells themselves but also boosts the cytotoxic behavior of infiltrating immune cells [62].

The effect of STAT5 on the melanoma–immune cell interplay is just beginning to be unveiled, mainly via studies in NK cells. The data demonstrate that STAT5 is indispensable for NK cell survival. Conditional knockdown of STAT5 in NK cells led to an almost entire loss of this cell population in transgenic mouse models. Subsequent intravenous injections of these mice with B16F10 melanoma cells increased tumor cell infiltration in the lungs when compared to the control groups [63]. In accordance with the results described above, Sathe et al. reported that an IL-15/JAK1/STAT5 pathway supports the generation of NK cells via transactivation of the antiapoptotic protein MCL1, and that *MCL1* conditional knockdown in NK cells in mice led to multiorgan metastases upon transplantation of melanoma tumors [64]. Other investigations revealed that forced expression of antiapoptotic *BCL2* rescues survival of STAT5-deficient NK cells [65]. Nevertheless, the presence of NK cells expressing high levels of BCL2, but lacking STAT5 was not sufficient to control tumor growth. As this study shows, lowering STAT5 levels in NK cells caused exacerbation of tumor growth in vivo, irrespective of the ability of NK cells to recognize and eradicate tumor cells [65]. They further demonstrated that upregulation of perforin, granzymes, and IFNγ by STAT5 is the key to the enhancement of NK cell cytotoxicity against melanoma [65]. In addition to NK cells, a STAT5-induced cytotoxic potential of immune cells towards skin cancer has also been reported for CD8+ T cells. In particular, genetic modification of CD8+ effector T cells with a constitutively active form of STAT5 gives them high efficiency for host colonization after adoptive transfer and transforms them into long-lived antigen-responsive cells. Upon transfer into melanoma-bearing hosts, these cells accumulate in the tumor site, become activated by tumor antigens, and express the cytolytic factor granzyme B, resulting in tumor regression [66]. It was recently shown that malignant melanoma cells induce inhibition of STAT5 signaling on cytotoxic NK and CD8+ T cells to achieve immune evasion and tumor progression. This effect is mediated by the melanoma-secreted dickkopf WNT signaling pathway inhibitor 2 (DKK2) [67].

**Figure 3:**
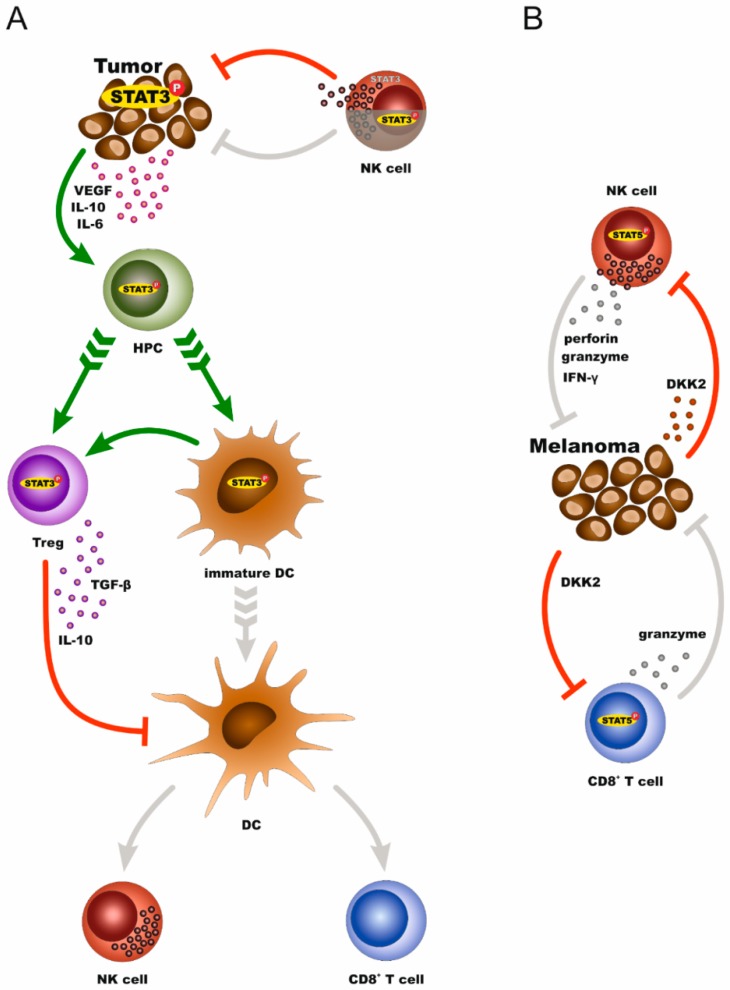
STAT3 and STAT5-mediated crosstalks between melanoma and immune cells create an immunosuppressive network that propels metastatic progression. (**A**) STAT3-expressing tumors secrete factors that upregulate STAT3 in hematopoietic progenitor cells (HPCs). This results in the accumulation of Tregs and immature dendritic cells in the tumor microenvironment, which further inhibit the maturation of these cells. Due to the lack of mature dendritic cells, the tumor-attacking CD8+ T cells and NK cells cannot be stimulated. NK cells with inactive STAT3 can repress tumors, while STAT3 activation in NK cells blocks their tumor-lysing properties. (**B**) STAT5-activated NK cells are effective against melanoma cells. This property is suppressed by secretion of DKK2 from melanoma cells (see main text for details).

In conclusion, STAT3 establishes a reciprocal relationship between melanoma cells and immune cells in favor of tumor immune evasion. STAT3-expressing cancer cells dysregulate immune cells, while at the same time, immune cells with high levels of STAT3 are unable to target melanoma cells. In contrast, STAT5 expression in tumor-attacking immune cells supports antitumor cytotoxicity (Figure 3). These sophisticated interactions between melanoma and immune cells via STAT proteins (and perhaps other factors) could provide a precocious basis towards interpreting the associations between melanoma and co-occurrent autoimmune conditions. Future studies are anticipated to shed more light on common mechanisms underlying melanoma and AD which could, at least in part, explain why these distinct pathologies of melanoma and autoimmunity appear, for example, in the same patients.

## 6. STAT3 and STAT5 Pathways in Inflammation and Melanoma-Associated Autoimmune Diseases

During inflammation, lymphoid and myeloid cells are recruited to the site of the lesion. By secreting cytokines, immune cells and macrophages control the development of inflammation. Effective and timely management of an inflammation is crucial for maintaining the immune system homeostasis. Any factor that activates inflammation or triggers immune reactions could lead to an autoimmune disease, whenever immune regulation of the inflammatory response fails to manipulate the situation appropriately. Thus, the root cause of most autoimmune diseases is the failure of the immune response to orchestrate the dynamics or magnitude of an inflammatory response, limit it to a relevant body site, and terminate it at the proper time [19]. The balance between Th17 cells, a subset of proinflammatory T helper cells which are characterized by IL-17 production, and Tregs that maintain tolerance to self-antigens is a key determinant in the development of an AD, and the signals that induce Th17 differentiation from CD4+ T cells inhibit Treg differentiation [68]. STATs affect inflammatory disease since they control the development of hematopoietic cells that regulate inflammation and mediate the responses of target cells to inflammatory cytokines [69]. For instance, STAT members, such as STAT1 and STAT3, are highly interlinked with NF-κB [70,71]. Not only does STAT3 respond to NF-κB-induced IL-6, a critical mediator of the acute phase response in inflammation [72], but it also physically interacts with this transcription factor to co-regulate inflammatory gene targets [71]. However, as the damage response persists, chronic activation of STAT proteins leads to inflammatory, idiopathic, and autoimmune conditions [72]. In line with this, *STAT3* gain-of-function mutations can cause early-onset lymphoproliferation and autoimmunity [73] and have been associated with Th17 hyperactivation [74]. STAT5 signaling is also involved in autoimmunity. Patients with STAT5B deficiency have decreased numbers of Treg cells and exhibit immunological aberrations, whereas most of them suffer from severe eczema and AD [49]. Herein, we briefly summarize our current knowledge of how STAT3 and STAT5 are implicated in inflammatory and autoinflammatory conditions as well as in autoimmune diseases. The effect of STAT3 and STAT5 on autoimmune diseases with a demonstrated link to melanoma [2] is described in Section 6.1 to Section 6.8 and depicted in Table 1. STAT3/STAT5-related inflammatory and autoimmune diseases with a still understudied effect on melanoma incidence are also mentioned (Section 6.9, Section 6.10, Section 6.11 and Section 6.12).

### 6.1. Peripheral Neuropathy

Peripheral neuropathy is an AD of paraneoplastic etiology, which may appear as melanoma comorbidity. It has been attributed to an immune response directed against antigens in the tumor that subsequently cross-react with the same or similar epitopes in the nervous system [75]. First evidence associates neuropathic pain with an activated JAK/STAT3 signaling pathway [76]. The exact role of STATs in this condition is yet to be determined.

### 6.2. Type 1 Diabetes Mellitus

Type 1 diabetes (T1D) is a chronic AD causing immune-mediated loss of pancreatic β cells. The key mediators of β cell destruction are the CD4+ and CD8+ T cells. Treg cells are also important for the maintenance of immune tolerance and prevention of autoimmunity. STAT3 is activated in T cells of T1D patients and favors their resistance to suppression [77]. It also influences diabetes by altering pancreatic cells development. This was shown in a study where an activating STAT3 mutation caused premature differentiation of stem cells into cells of the pancreatic endocrine lineage [78]. Similar to STAT3, STAT5 activation has been correlated with T1D, since persistent STAT5 phosphorylation characterizes monocytes of individuals with or at-risk for T1D and is suspected of altering the epigenetic regulation of inflammatory response genes [79].

### 6.3. Rheumatoid Arthritis

Rheumatoid arthritis (RA) is a chronic disorder manifested with continuous inflammation, swelling, destruction, and pain in multiple joints. Th17 cells are the pathogenic culprit in RA, while Treg cells suppress RA. STAT3 and STAT5 knockdown in RA demonstrated that STAT3 increases Th17 but decreases Treg proportions, whereas STAT5 has the opposite effects on these cell populations [80], providing insights that STAT5 counteracts STAT3 activity on the reciprocal balance of Th17 and Tregs in the context of RA. In addition, STAT3 induces factors that lead to inflammation and osteoclast genesis, while its conditional knockout in mice blocks joint inflammation and destruction and offers resistance to collagen-induced arthritis [81]. Consistently, STAT3 phosphorylation and subsequent upregulation of its downstream gene targets are discriminative for CD4+ T cells of RA patients [82]. It is possible that STAT5 counteracts STAT3 in the context of RA via exerting opposite effects in the Th17/Treg balance, which is crucial in the pathogenesis of this disease [83].

### 6.4. Psoriasis

Psoriasis is a chronic autoimmune disease characterized by patches of abnormal skin, which are typically red, dry, itchy, and scaly. Its most prevalent type is psoriasis vulgaris. It is considered a T cell-mediated AD modified by genetic susceptibility and environmental stimuli. Th17 lymphocytes, which differentiate from naïve T cells upon IL-6 induction, play a central role in its pathogenesis, and recent evidence suggests that inflammatory circuits are established among Th1 and Th17 cells and keratinocytes, promoting psoriasis. This leads to the hypothesis that STAT3 and STAT5, which regulate Th17, are possibly associated with psoriasis. Indeed, STAT3 activation has been observed in the skin of psoriatic patients [84]. Consistently, transgenic mice, in which keratinocytes express a constitutively active form of STAT3, develop psoriasis-like skin lesions. Topical administration of STAT3 inhibitor improved psoriatic lesions in both, these mice and in clinical patients [85]. The role of STAT5 in psoriasis needs to be assessed.

### 6.5. Autoimmune Pancreatitis

Autoimmune pancreatitis is a unique form of chronic pancreatitis initially reported in 1995. It exhibits characteristic histological features, frequent elevations of serum IgG4 antibodies, and a predictable response to steroid therapy. Potential long-term sequelae include pancreatic duct stones and malignancy [86]. A recent report describes a case of acute pancreatitis that presented in a patient with a loss-of-function STAT3 mutation [87]. The involvement of STATs in this newly-identified autoimmune condition remains to be elucidated.

### 6.6. Autoimmune Aplastic Anemia

Aplastic anemia (AA) is a non-malignant blood disorder, caused by immune destruction of hematopoietic and progenitor cells, which leads to a failure of bone marrow to sustain blood production. As a result, the patients develop pancytopenia, evidenced by low levels of all blood cell types. Clonal hematopoiesis with the formation of a genetically distinct subpopulation of blood cells has been suggested as a basis of this disease, where clonal cells have acquired the ability of increased proliferation and immune escape [88]. *STAT5B* [88] and *STAT3* [89] gain-of-function mutations have been found in T cells of AA patients, while an IL-2/STAT5B pathway characterized patients’ Treg subpopulations [90]. Overall, these data link STATs with AA, although continued investigation into the mechanistic connections is necessary.

### 6.7. Hashimoto’s Encephalopathy

Hashimoto’s encephalopathy is a rare disease of autoimmune origin, characterized by high titers of antithyroid antibodies. It has been proposed that an abnormal function of the immune system causes neuronal inflammation, leading to impaired brain function. The condition co-exists with other ADs, such as T1D mellitus, systemic lupus erythematosus, and Sjögren syndrome [91]. Although the molecular mechanism of this rare disease remains elusive, a study showing that STAT5 programs a distinct subset of Granulocyte-macrophage colony-stimulating factor (GM-CSF)-producing T helper cells, which is essential for autoimmune neuroinflammation [92], could provide a potential link between STATs and this disorder. Future investigations could shed more light on possible associations of STATs with the etiopathology of encephalitis.

### 6.8. Inflammatory Bowel Disease

Inflammatory bowel disease (IBD) describes a group of chronic, relapsing, autoimmune diseases of complex etiology that become manifest at the small intestine and colon. It encompasses ulcerative colitis and Crohn’s disease, which differ in the location of inflammation within the gastrointestinal tract. Several cytokines that activate STAT3 drive IBD pathogenesis. Data obtained from IBD patients showed that STAT3 is activated in their inflamed colons. Intriguingly, expression of STAT3 in different immune cell populations exerts opposing effects on IBD. In particular, phosphorylated protein in T-cells contributes to colitis, while its activation in myeloid cells (neutrophils and macrophages) and enterocytes is protective against colitis. In mouse models of IBD, several proinflammatory cytokines, such as IL-2, IL-6, IL-15, IL-21, IL-23, IL-17, and IL-18, have detrimental effects, whereas anti-inflammatory cytokines, including IFNα/β, IL-10, IL-11, and IL-22, are beneficial. STAT3 targeting with small molecule inhibitors ameliorates IBD in vivo and is under consideration as a new approach to IBD management, particularly in patients that are refractory to current therapies [72]. However, given the opposing functions of STAT3 on different immune cell types, targeting should be directed towards the specific cell population which promotes IBD, without jeopardizing those immune cells that protect from disease [93].

STAT5 has rather anti-inflammatory properties both in the intestinal epithelium and in adaptive immune cells and has been shown to oppose STAT3 functions in the context of IBD. A GH/STAT5 axis is protective against colitis in animal models. On one side, STAT5 is critical for the proliferation of intestinal epithelial stem cells and regeneration of the crypt epithelium. STAT5B-deficient mice are particularly susceptible to chemically induced colitis, a fact that was attributed to apoptosis of epithelial cells and damage of the mucosal barrier. In agreement, activated STAT5 promotes mucosal wound healing, increases intestinal epithelial stem cell proliferation, accelerates crypt regeneration, and confers resistance to intestinal injury [95,96]. Lack of active STAT5 predisposes to *Clostridium difficile* infection-induced colitis [97]. Otherwise, STAT5 regulates T cell differentiation, especially Treg cells. In addition, IL-2 signaling via STAT5 limits Th17 in favor of Treg development. This is opposed to STAT3 function, which induces Th17 differentiation and negatively controls Treg differentiation. The balance between Tregs and effector T cells is key in IBD suppression. Moreover, GM-CSF-induced STAT5 signaling protects dendritic cells from apoptosis, in contrast to the apoptotic effects of an IL-21/STAT3 axis in these cells [94].

### 6.9. Asthma

Asthma is a condition characterized by inflammation, which leads to hyperresponsiveness of the airways and remodeling of the airway wall. Airway inflammation and remodeling is accompanied by STAT3 activation and by increased levels of Th2- and Th17-type cytokines in the lung. STAT3 regulates immune cell recruitment, specifically Th2 cells, during allergic inflammation. Targeted STAT3 depletion in the airway epithelium prevents house dust mite-mediated allergic inflammation and airway hyperresponsiveness. Interestingly, other studies suggest that Th2 cells respond to STAT3 activation differentially, depending on their location in the lung [72]. In a similar manner, in animal models of asthma, the STAT5 pathway-regulates lymphocyte proliferation induced by ovalbumin [98], while a GM-CSF/STAT5 axis enhances survival of lung granulocytes [99]. Observations on a large cohort of hospitalized asthma patients revealed an increased cancer incidence, which, however, was highly cancer type-dependent. These patients presented an increased risk for stomach and colon cancer but decreased for endometrial cancer and melanoma [100]. Further studies which could also include the population of outpatient asthma patients are needed to investigate the potential link between asthma and several cancer types.

### 6.10. Cachexia and Polymyositis

Cachexia is an inflammation-driven metabolic syndrome, where impaired regulation of the balance between anabolism and catabolism results in the loss of adipose tissue and skeletal muscle, eventually leading to muscle atrophy. Mechanistic studies have shown that STAT3 phosphorylation in response to overexpression of its upstream cytokine IL-6 is associated with this condition [72]. In a similar manner, levels of phosphorylated STAT3 and cytokine IL-22 have been found elevated in muscle tissues of patients with polymyositis, an idiopathic inflammatory myopathy that causes muscle weakness [101]. Several known STAT3 inhibitors that directly target hyperphosphorylated STAT3 also reduced markers of cachexia in cell culture models and antagonized catabolic signaling in mice, supporting the concept that STAT3 is a valid target for cachexia treatment [72]. No study has correlated STAT5 and polymyositis thus far. An association between cachexia and melanoma has been suggested based on mice studies. In detail, B16F10 melanoma-bearing mice depicted skeletal muscle and epididymal fat mass reduction, muscle strength loss, and locomotor activity impairment, associated with elevated IL-6 levels in the plasma [102]. Further studies are needed to investigate if these effects are mediated by STATs.

### 6.11. Fibrosis

Tissue fibrosis is an inflammatory condition which is caused upon dysregulation of the tightly-controlled process of wound healing and is characterized by overproduction of the extracellular matrix (ECM) and excess matrix contraction. STAT3 drives wound healing following inflammation and contributes to fibrosis by inducing production of ECM or by transactivating profibrotic metalloproteases, such as MMP-9. The blockade of STAT3 phosphorylation with small molecule inhibitors could ameliorate the development of pulmonary fibrosis in mice exposed to bleomycin [72]. In contrast to the profibrotic function of STAT3, STAT5 seems to protect from fibrosis, perhaps through counteracting STAT3. In particular, loss of STAT5 causes liver fibrosis through STAT3 activation [103]. It was recently shown that the dorsal skin of mice contains multiple and diverse lineages of fibroblasts. One of those lineages is responsible for the fibrotic response to injury and cancer-stroma formation. Ablation of this specific lineage diminished connective tissue deposition in wounds and reduced melanoma growth in vivo [104]. This study provides an initial link between melanoma and fibrotic injury, although further investigation is required to confirm this finding in the cancer patient population and to unveil the underlying mechanisms.

### 6.12. Systemic Lupus Erythematosus

Systemic lupus erythematosus (SLE) is a chronic, systemic AD that distinguishes itself by autoreactive lymphocytes and proinflammatory cytokine production. It can often lead to renal injury and multiple organ damage due to the formation of autoantibodies against nuclear antigens, cell surface, and serum proteins [105]. Both STAT3 and STAT5 are implicated in SLE etiopathology and have been found activated in immune cells of SLE patients [106,107]. In T cells of SLE patients, STAT3 and STAT5 synergistically transactivate IL-10 through epigenetic remodeling [108]. In the development of SLE, dysregulation of the Th17/Treg balance may play an important role and could be responsible for an increased proinflammatory response, especially in the active form of the disease [109,110]. In this context, SLE has been recently associated with an active IL-17/STAT3 axis, accompanied by higher Th17 cell numbers [111]. STAT3 inhibitors in combination with immunosuppressive drugs, improve SLE via restoration of this balance [105], while agents inhibiting STAT3 phosphorylation have shown efficacy in the treatment of SLE [106]. STAT5 is also implicated in SLE, since activated STAT5 upregulates the antiapoptotic targets Bcl-2 and Ki-67 in CD4+ T cells, perhaps providing them with a survival and proliferative advantage over Treg cells [112]. A large patient population-based, retrospective study recently highlighted a statistically significant association between SLE and melanoma [113]. Dreyer et al. also described that SLE tends to be positively associated with melanoma, although the trend was not statistically significant [114]. However, another meta-analysis study reported a decreased risk of melanoma in SLE patients [115]. Further exploration of the association between malignant melanoma and SLE is anticipated to shed more light on this issue.

## 7. Perspectives, Challenges, and Limitations Towards Translating STAT3/STAT5 Targeting to Therapeutic Solutions for Patients with AD and Melanoma

Recent studies converge to the revolutionary idea of targeting a single nodal molecule to manage two inter-related pathological conditions simultaneously [116,117,118]. In line with this notion, we evaluated whether STAT targeting can be considered for the management of melanoma and its associated autoimmune comorbidities. An advantage of this approach is that there is an already established arsenal of STAT3 and STAT5 inhibitors, which are currently under investigation for the treatment of cancer or autoimmune diseases, as reviewed in detail by Loh and colleagues [40]. This arsenal is being further enriched by indirect epigenetic modifiers of STAT proteins [70,119,120]. STAT3 inhibitors have demonstrated their efficacy in animal tumor models [121] and are currently under ongoing clinical trials for melanoma treatment (ClinicalTrials.gov Identifiers: NCT01904123, NCT03195699). In a similar manner, these inhibitory molecules are also efficient in treating several autoimmune diseases in vivo [85,106] and are now tested in a clinical anti-T1D setting (ClinicalTrials.gov Identifier: NCT02641522), which has been reported as melanoma-associated comorbidity. Moreover, it has been reported that cancer patients who exhibit cachexia may benefit from STAT3 inactivation, as it preserves fat and tissue mass and rescues cachectic phenotypes, in addition to eliminating tumors [122]. Another approach for simultaneous disease management could be achieved through nutritherapeutic strategies. Several dietary compounds are potent inhibitors of STAT3 regulatory networks [123,124]. These nutriceuticals could have potential applications in patients with chronic ADs, in the sense that their long-term use as dietary supplements might not only mitigate AD symptoms, but also reduce the cancer risk. Hence, STAT3 inhibitors may hold therapeutic promise as multipurpose drugs for the treatment of melanoma in conjunction with associated ADs. Due to its roles in cancer and immunity, STAT5 is also a proposed therapeutic target [125]. Studies in AD animal models with transplanted melanoma tumors versus immunocompetent controls will not only provide proof-of-concept about the feasibility of using agents such as STAT inhibitors against different diseases but might also establish an experimental approach to facilitate insights into the complex interactions between melanoma and immune cells.

One challenge regarding the proposed approach remains, namely which melanoma–immune cell interactions would be suitable for modulation to achieve dual targeting of these diseases without reducing the beneficial effects of other immune cell populations. Following this direction, it is intriguing to hypothesize that a possible interplay among melanoma, Th17, and Treg cells could be manipulated towards this goal. In detail, it has been recognized that an unbalanced Th17/Treg response, on the one hand, mediates several melanoma-associated Ads, such as psoriasis, IBD, RA, and SLE, and on the other hand, contributes to carcinogenesis mainly via propelling chronic inflammation [126]. Interestingly, STAT3 and STAT5 emerge as fine-tuners of the Th17/Treg balance, since STAT3 is responsible for Treg cells and STAT5 mediates Th17 differentiation [126]. Given that Th17 [127] and Tregs [128] have been found dysregulated during melanoma progression, it is appealing to postulate that disturbance of the Th17/Treg homeostasis may favor melanoma cell proliferation in a STAT3/STAT5-dependent manner. Future studies could shed more light on this issue.

Targeting of the interplay between melanoma cells and NK cells poses as another challenge. While STAT3 inhibition reactivates NK cells against melanoma, STAT5 inhibition suppresses tumor cytotoxic activities of NK cells. A hypothetical explanation is that NK cells could be particularly sensitive to a still understudied equilibrium between STAT3 and STAT5. Such a scenario poses limitations to the straightforward inhibition of either STAT3 or STAT5 in NK cells and necessitates elucidating how the STAT3/STAT5 ratio is regulated and what is its contribution to the melanoma–NK cells interplay. In NK cells, STAT3 inhibition leads to STAT5 activation, and a delicate balance exists between STAT3-mediated suppression and STAT5-induced activation of NK-cell cytotoxicity [62]. It has been hypothesized that this inverse correlation between STAT3 and STAT5 could serve as a regulator of autoimmunity, given that both factors respond to the same cytokines. In particular, IL-15-induced STAT3 activation could counteract the IL-15-STAT5-mediated NK-cell cytotoxicity to prevent autoimmunity [62]. A detailed understanding of the mechanisms governing transactions between melanoma and immune cell populations could catalyze the development of anticancer strategies aiming at increasing the potential of killer cells while suppressing self-destruction.

Along with hopes, targeting of STAT factors has also raised skepticism, since its pleiotropic effects in non-tumor tissues are predicted to potentiate the risk of adverse events [129]. Indeed, STATs are points of convergence of several signal transduction pathways that affect diverse processes [130], and as such, their inhibition will have multiple effects, some of which may be manifested as side effects in nontumor tissues. However, this disadvantage can theoretically turn into an advantage in the event of multimorbid cancer patients, since in these patients systemic administration of inhibitors against pleiotropic molecules that are commonly activated in melanoma and associated comorbidities could, in the long run, simultaneously manage lesions. In this case, the side effect at a site outside the tumor could be proven beneficial if it actually counteracts a co-existing AD. Such directions could inspire next-generation therapeutics, especially in melanoma patients whose pre-existing conditions refrain them from getting benefit from current immunotherapy regimens.

## 8. Conclusions

Inflammation and autoimmune disorders emerge as comorbidities in melanoma, providing hints that deregulation in common processes may govern all-three diseases. Crosstalks are established among melanoma cells and several subpopulations of immune cells, reciprocally modifying their behavior. Molecular drivers of these crosstalks, such as STAT3 and STAT5 signaling pathways, could be targeted to manipulate interactions between melanoma cells and immune cells, to develop therapeutics that coordinately regulate skin cancer and inflammatory and/or autoimmune conditions. In this review, we (a) state that the high-risk patients with a non-competent immune system (i.e., those with a pre-existing AD or transplant organ recipients) constitute a special population which requires personalized management with novel therapeutics that are able to both kill tumor cells and suppress immune system responses, (b) evaluate if such an approach is feasible, based on the molecular commonalities between tumors, inflammation, and autoimmune disease, and (c) estimate STAT targeting as a representative example, although targeting of additional, probably still undiscovered molecules, may also exert a similar multipotent effect. Immunotherapeutics have taken center stage and emerge as the blockbusters of pharmacopoieia. Their favorable effects on cancer patients keep therapeutic hopes high. However, the use of cancer immunotherapy on patients with associated autoimmune disease could lead to life-threatening side effects. In contrast, drugs with an inherent ability to cause immune suppression which could theoretically counteract an overactive immune system might constitute a more suitable option in the context of personalized therapy. To this end, selected next-generation therapeutics, e.g., STAT inhibitors, may have the potential to shape the first line of treatment for these patient populations in the future.

## Figures and Tables

**Table 1:** Effects of STAT3/STAT5 in autoimmune diseases associated with melanoma (conditions are listed by decreasing prevalence rate in melanoma patients).

AD	STAT3 Input	STAT5 Input	STAT3 Inhibition	STAT5 Inhibition	Refs
Peripheral Neuropathy	activated JAK/STAT3 signaling pathway	n.d.	n.d.	n.d.	[75,76]
Type 1 Diabetes Mellitus	activated in T cells, premature differentiation of stem cells into cells of the pancreatic endocrine lineage	STAT5 phosphorylation in monocytes	therapeutic	n.d.	[77,78,79]
Rheumatoid Arthritis	activated in CD4+ T cells, increase of Th17/Treg ratio, induction of inflammatory and osteoclastogenic factors	decrease of Th17/Treg ratio	therapeutic	n.d.	[80,81,82,83]
Psoriasis	STAT3 activation in the skin	n.d.	therapeutic	n.d.	[84,85]
Autoimmune Pancreatitis	STAT3 loss-of-function mutation	n.d.	n.d.	n.d.	[86,87]
Autoimmune Aplastic Anemia	STAT3 gain-of-function mutations in T cells	IL-2/STAT5B active in Tregs, STAT5B gain-of-function mutations in T cells	n.d.	n.d.	[88,89,90]
Hashimoto’s Encephalopathy	n.d.	activation of neuroinflammation-promoting T helper cells	n.d.	n.d.	[91,92]
Inflammatory Bowel Disease	STAT3 activation in T-cells contributes to colitis, STAT3 activation in myeloid cells protects from colitis	proliferation of intestinal epithelial stem cells, regeneration of the crypt epithelium, regulation of Th17/Treg balance, protection of dendritic cells from apoptosis, mucosal wound healing, resistance to intestinal injury	therapeutic	n.d.	[72,93,94,95,96,97]

n.d.: not determined.
